# Health Assessment and Seroepidemiologic Survey of Potential Pathogens in Wild Antillean Manatees (*Trichechus manatus manatus*)

**DOI:** 10.1371/journal.pone.0044517

**Published:** 2012-09-12

**Authors:** Kathryn Sulzner, Christine Kreuder Johnson, Robert K. Bonde, Nicole Auil Gomez, James Powell, Klaus Nielsen, M. Page Luttrell, A. D. M. E. Osterhaus, A. Alonso Aguirre

**Affiliations:** 1 Wildlife Health Center, School of Veterinary Medicine, University of California Davis, Davis, California, United States of America; 2 Sirenia Project, United States Geological Survey, Gainesville, Florida, United States of America; 3 Sea to Shore Alliance, St. Petersburg, Florida, United States of America; 4 Canadian Food Inspection Agency, Nepean, Ontario, Canada; 5 Southeastern Cooperative Wildlife Disease Study, Department of Population Health, College of Veterinary Medicine, University of Georgia, Athens, Georgia, United States of America; 6 Erasmus Medical Centre, Rotterdam, The Netherlands; 7 Smithsonian-Mason School of Conservation, Front Royal, Virginia, United States of America; 8 Department of Environmental Science and Policy, George Mason University, Fairfax, Virginia, United States of America; Université Catholique de Louvain, Belgium

## Abstract

The Antillean manatee (*Trichechus manatus manatus*), a subspecies of the West Indian manatee, inhabits fresh, brackish, and warm coastal waters distributed along the eastern border of Central America, the northern coast of South America, and throughout the Wider Caribbean Region. Threatened primarily by human encroachment, poaching, and habitat degradation, Antillean manatees are listed as endangered by the International Union for the Conservation of Nature. The impact of disease on population viability remains unknown in spite of concerns surrounding the species’ ability to rebound from a population crash should an epizootic occur. To gain insight on the baseline health of this subspecies, a total of 191 blood samples were collected opportunistically from wild Antillean manatees in Belize between 1997 and 2009. Hematologic and biochemical reference intervals were established, and antibody prevalence to eight pathogens with zoonotic potential was determined. Age was found to be a significant factor of variation in mean blood values, whereas sex, capture site, and season contributed less to overall differences in parameter values. Negative antibody titers were reported for all pathogens surveyed except for *Leptospira bratislava*, *L. canicola*, and *L. icterohemorrhagiae*, *Toxoplasma gondii*, and morbillivirus. As part of comprehensive health assessment in manatees from Belize, this study will serve as a benchmark aiding in early disease detection and in the discernment of important epidemiologic patterns in the manatees of this region. Additionally, it will provide some of the initial tools to explore the broader application of manatees as sentinel species of nearshore ecosystem health.

## Introduction

The Antillean manatee (*Trichechus manatus manatus*), a subspecies of the West Indian manatee, is an herbivorous, aquatic mammal restricted to warm coastal waters and inlets along Central America, the northern coast of South America, and the Caribbean [1], [2], [3]. With populations continuing to decline, the Antillean manatee is currently listed as endangered by the International Union for the Conservation of Nature (IUCN) [4]. The manatees inhabiting the waters of Belize are thought to serve as a vital source population for manatee populations occupying the neighboring coasts of Mexico, Guatemala, and Honduras [2]; thus, ongoing efforts to conserve this population are crucial. Unfortunately, low recruitment, due in part to a lengthy calving interval, may not be adequate to maintain population viability in the face of mounting anthropogenic threats [2], [5], [6]. Based on preliminary assessments, two-thirds of all manatee mortalities in Belize can be traced to habitat loss, perinatal death, and human activities including poaching, trauma suffered from collisions with watercraft, and fatalities from fishing equipment [2].

Current strategies to protect manatees in Belize are focused on mitigating anthropogenic pressures but do not effectively address the remaining one-third of manatee deaths resulting from unspecified causes [2]. In particular, little is known about the impact of disease at the population level or on the stability of sirenian populations elsewhere [2], [6]. Although manatees are thought to be fairly resistant to natural disease [5], [7], [8], [9], shifts in the aquatic environment brought on by climate change, sea level rise, human encroachment, habitat destruction, and pathogen pollution at the land-water interface, may make this nearshore species increasingly susceptible to infectious agents [10], [11], [12], [13]. Sudden changes in manatee health may signify a larger environmental disturbance at play, as has been demonstrated with brevetoxin-related epidemics [14], [15]. Manatees may therefore be useful sentinels of the surrounding ecosystem [7], [10], [16], illustrating the broader application of health research in this species.

In order to evaluate baseline health in Belize’s manatee population, a collaborative, multi-agency health assessment initiative was launched in 1997, complementing objectives outlined in the Belize’s Manatee Recovery Plan [2]. Between 1997 and 2009, blood samples and other health-related data were collected from 115 wild Antillean manatees captured and examined in southern Belize. The present study was integral to this larger manatee health initiative by fulfilling two primary objectives: The first was to establish normal hematologic and biochemical reference intervals for the manatees of this region; the second was to describe the seroepidemiology of eight potential pathogens in the wild manatee population of southern Belize. The pathogens of interest were selected for their zoonotic capabilities and for their association with morbidity and mortality events in other marine mammals.

## Materials and Methods

### Ethics Statement

Approval for this project was granted to lead scientists from the following governmental and non-governmental agencies and programs: The Coastal Zone Management Authority and Institute’s (CZMAI), *Manatee Project*, under the jurisdiction of Belize’s Ministry of Agriculture, Fisheries, and Co-operatives; Belize’s National Manatee Working Group; Ecohealth Alliance; and Sea to Shore’s Manatee Conservation Program in Belize. The Institutional Animal Care and Use Committees of Ecohealth Alliance and U.S. Geological Survey, Southeast Ecological Science Center, permitted authorization for research, including manatee capture and sampling protocols. Data and samples were collected under research permits issued by the Belize Forest Department, Ministry of Natural Resources, and the U.S. Fish and Wildlife Service permit number M79 1721-4 issued to the U.S. Geological Survey, Sirenia Project.

### Manatees and Sample Collection

**Figure 1:**
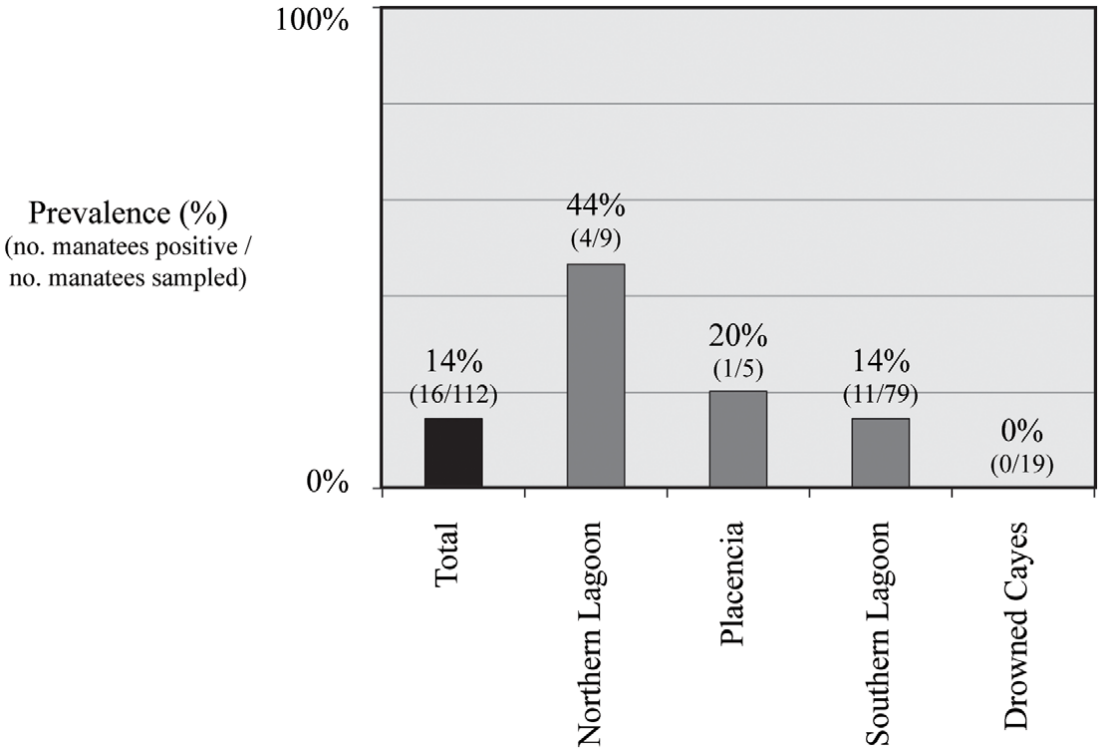
Seroprevalence (%) of exposure to *Leptospira bratislava* in the sample population as a whole and by capture site in wild Antillean manatees *(Trichechus manatus manatus)* in Belize between 1997 and 2009.

Between 1997 and 2009, blood samples were collected opportunistically from 115 apparently healthy, wild Antillean manatees in Belize. With few exceptions, data collection took place biannually, typically occurring during alternating wet and dry-seasons (i.e., May/June – November and December – April/May, respectfully) [17], [18]. Captures occurred in, or around the periphery of, one of four primary sites: Southern Lagoon in Gales Point Wildlife Sanctuary (17.20532°N, 88.33643°W); Northern Lagoon (17.35481°N, 88.33025°W); Placencia Lagoon (16.53184°N, 88.37703°W); and the Drowned Cayes area (17.48281°N, 88.09765°W) (Fig. 1). For the purposes of this study, manatees captured in Western, Quashie Trap, Buttonwood, and Sapodilla lagoons, and those captured along the coast near Mullins River mouth were included in the Southern Lagoon subpopulation since travel among these sites is common.

A standard capture technique was employed [19], [20] in which manatees were approached by boat in near shore water of 1–1.5 meters in depth. A large, 13 cm stretch-mesh, nylon net (152 m long, 6 m deep) was then lowered into the open water surrounding the target manatee [19]. The net was then collapsed, and individuals, once restrained, were carefully transported to shore or brought onto the deck of the capture boat for examination. A physical exam was performed on each manatee by a trained veterinarian or biologist under veterinary supervision prior to sample collection [21]. The sex of the individual was determined by assessment of dimorphism of the genital-anal distance [20], [22], [23]. Age classification was based on standardized total straight length measurements specific to Antillean manatees [24]: adult (>225 cm); subadult (176–225 cm); and calf (<176 cm). Detailed morphometrics were recorded, and subcutaneous fat thickness measurements were taken with an ultrasound. Vital signs were recorded throughout the holding time to monitor for signs of distress [25].

Following the recommended technique for venipuncture in manatees [20], [26], blood samples were obtained from the brachial vascular bundle, located on the medial aspect of the flipper. Blood draws were performed using an 18 or 21 gauge, 1.5 inch needle attached to an extension set equipped with a Luer® adapter in a Vacutainer® collar (Becton-Dickson (BD), Franklin Lakes, New Jersey, USA). Approximately 20–40 ml of whole blood were collected from each animal into potassium EDTA anticoagulant Vacutainer® blood tubes (BD, Franklin Lakes, New Jersey, USA) for complete and differential blood cell counts. An additional 60–80 ml of whole blood were obtained for serologic and biochemical analysis; this quantity was divided into plain red top or tiger top serum separator Vacutainer® tubes for serum (BD, Franklin Lakes, New Jersey, USA) and green top lithium heparinized Vacutainer® tubes for plasma (BD, Franklin Lakes, New Jersey, USA). All blood samples were placed on ice packs at 4°C immediately after venipuncture. Samples intended for serology or biochemistry were centrifuged within eight hours of collection. Following centrifugation, the serum (supernatant) and plasma from these tubes was transferred to either one or four ml aliquots depending on total yield.

Once collected, blood samples were brought to the Belize Medical Associates Laboratory (BMAL) (Belize Medical Associates, 5791 St. Thomas Street, Belize City, Belize), where they were either processed for hematology and biochemistry analysis within 12–24 hours of collection or transferred to a −20° freezer. Sera and plasma to be archived were transported to Florida under the Convention on International Trade in Endangered Species (CITES) permit authority and stored in a −80°C freezer at the U.S. Geological Survey, Southeast Ecological Science Center in Gainesville, Florida (USGS SESC, Gainesville, FL). Subsamples of serum and plasma were shipped to the University of Florida, College of Veterinary Medicine, Clinical Pathology Laboratory (UFPL) (University of Florida Pathology Laboratories, Gainesville, FL) for additional biochemistry analysis.

### Laboratory Analysis

Complete and differential blood cell counts performed at BMAL included the following parameters: packed cell volume (PCV); white blood cell count (WBC); red blood cell count (RBC); hemoglobin (Hb); mean corpuscular volume (MCV); mean corpuscular hemoglobin (MCH); mean corpuscular hemoglobin concentration (MCHC); and platelets (PLT). White blood cell differentials were performed for granulocytes (i.e., neutrophils, eosinophils, and basophils) and for agranulocytes (i.e., lymphocytes and monophils). Differentials were converted to absolute cell counts for statistical analysis.

At the start of the study, complete serum biochemistries were performed at BMAL. After 2003, additional biochemistry profiles were performed on plasma samples at UFPL. For quality control purposes, a blind test was run to compare serum biochemistry results from BMAL with plasma biochemistry results from UFPL in individuals from which both serum and plasma samples were obtained. With the exception of minor disparities involving three analytes: alanine aminotransferase (ALT), aspartate aminotransferase (AST), and triglycerides, there were no significant differences in biochemistry results between the two laboratories or between serum and plasma samples from the same individual. To maintain consistency in reference interval determination for ALT, AST, and triglycerides, serum samples continued to be processed at BMAL for these three analytes.

Plasma biochemistry profiles conducted at UFPL were performed on a Hitachi 911™ Chemistry Analyzer (Boerhinger Mannheim/Roche Applied Science, Indianapolis, Indiana, USA) using reagents and technique described elsewhere [27]. The biochemistry profile included the following blood analytes: glucose; blood urea nitrogen (BUN); creatinine; alkaline phosphatase (ALP); gamma-glutamyl transferase (GGT); cholesterol; total protein (TP); albumin (ALB); potassium; magnesium; chloride; sodium; calcium; phosphorous; total bilirubin; direct bilirubin; amylase; lipase; lactate dehydrogenase; creatinine kinase phosphokinase; serum amyloid A; and iron.

Pathogens selected for serologic testing included the following bacterial, parasitic, and viral agents listed respectively*: Brucella* spp. and *Leptospira interrogans* spp.; *Neospora caninum*, *Sarcocystis neurona*, and *Toxoplasma gondii;* and avian influenza virus type A (AIV), morbillivirus, St. Louis encephalitis virus (SLEV), Western equine encephalitis virus (WEEV), and West Nile virus (WNV). None of the pertinent serologic tests available have been validated in manatees. In view of this, assays for which positive controls have already been established in other terrestrial or marine mammals were chosen as the next best alternative.

A fluorescence polarization assay was performed to test for antibodies to *Brucella* spp. (Klaus Nielsen, Canadian Food Inspection Agency, Nepean, Ontario, Canada) using positive controls of bovine origin and corresponding methods to identify seropositive animals described elsewhere [28]. Detection of antibodies to the following panel of *L. interrogans* serovars: *L. bratislava*; *L. canicola*; *L. gryppotyphosa*; *L. hardjo*; and *L. icterohemorrhagiae* was achieved using a modified aggluntination test (MAT) (positive titer >1∶100), (California Animal Health & Food Safety Laboratory System (CAHFS) - Davis Laboratory, University of California, Davis, CA).

An indirect fluorescent antibody test (IFAT) was run for detection of antibodies to *N. caninum, S. neurona*, and *T. gondii*, (Department of Pathology, Microbiology and Immunology, School of Veterinary Medicine, University of California, Davis, CA). In order to perform the IFAT on manatee sera, fluorescein isothiocyanate (FITC) - labeled goat anti-mouse IgG Fc - specific secondary antibody against purified manatee IgG was produced at the University of Florida (Interdisciplinary Center for Biotechnology Research Hybridoma Core Laboratory, University of Florida, Gainesville, FL). Results for *T. gondii* were further verified with a latex agglutination test (LAT). In the absence of serological test validation for *T. gondii* in manatees, we relied on published studies and laboratory recommendations as a guide for selecting cut-off titers for the IFAT (≥ 1∶320) [29] and LAT (≥ 1∶40) [30], [31], [32].

Serologic testing for morbillivirus was accomplished using a viral neutralization (VN) assay against canine distemper virus (CDV) and against porpoise and dolphin morbilliviruses (PMV/DMV) (A.D.M.E Osterhaus, Department of Virology, Erasmus Medical Centre, Rotterdam, The Netherlands). A neutralization antibody titer response ≥ 10 was considered positive [33]. A commercial blocking enzyme-linked immunosorbent assay (FlockCheck AI MultiS-Screen Antibody Test Kit, IDEXX Laboratories, Westbrook, ME), validated with serum samples from ferrets that were experimentally infected with AIV, was performed to assess serum antibodies to AIV type A (Department of Population Health, College of Veterinary Medicine, University of Georgia, Athens, GA). Absorbance values were read with a Bio-Rad Benchmark Microplate Reader (Hercules, California, USA) at 650 nm, and serum sample to negative control (S/N) absorbance ratios were calculated for each sample. Samples with S/N values greater or equal to 0.50 were considered negative for the presence of antibodies to AIV, and samples with S/N values less than 0.50 were considered positive. Manatee sera samples were screened for SLEV, WEEV, and WNV with an Enzyme immunoassay (EIA) (Center for Vectorborne Disease Research, University of California, Davis, CA) using biotin labeled goat anti-mouse IgG Fc-specific secondary antibody against purified manatee IgG prepared at the University of Florida (Interdisciplinary Center for Biotechnology Research Hybridoma Core Laboratory, University of Florida, Gainesville, FL). Samples were considered positive if a ratio of greater than two was observed based on the average reading of two positive wells/one negative well.

### Statistical Analysis

Statistical analyses were performed for hematology, biochemistry, and serology data using the statistical software program, STATA 11 (StataCorp, College Station, Texas). Basic descriptive measures and graphical summaries including mean, median, standard deviation, and histograms were used to evaluate the distribution of each hematologic and biochemical variable. Normality was assessed with the Shapiro-Wilks W test for normality [34], along with standard methods to assess skewness and kurtosis. The same procedures were performed on the residuals for each variable [35]. Corresponding plots (e.g. residual versus fitted, residual versus predictor, kernel density, and histogram) were also evaluated to further confirm which variables approximated a Gaussian distribution and verify the decision to remove certain values designated as outliers for each blood parameter [36]. Several variables (e.g. RBC, WBC, lymphocyte count, glucose, BUN, creatinine, and ALT) were log-transformed to better approximate a Gaussian distribution. The log-transformed variables were then re-examined by the previous methods to ensure that they satisfied the criteria and met the assumptions of the statistical analyses chosen.

A two-way analysis of variance (ANOVA) was used to evaluate sources of variability in blood values in relation to sex and age. In examining differences in blood parameters by capture site, both Placencia and Northern lagoons were excluded from the analysis of all hematologic parameters due to inadequate sample size (n<5); this was also the case for several biochemical parameters. Consequently, when comparisons were restricted to two capture sites (e.g. Southern Lagoon and Drowned Cayes), an unpaired t-test was used to assess differences in mean parameter values. When more than two capture sites met the sample size requirements for a given blood parameter, a one-way ANOVA was performed. An un-paired t-test was used to assess differences between group means across wet and dry seasons. For comparisons involving blood parameters that did not approximate a Gaussian distribution, even if log-transformed (i.e., monocytes, basophils, and eosinophils), a Kruskal-Wallis one-way analysis of variance was conducted. To avoid violating assumptions of independence, only the first adequate blood sample (i.e., non-hemolyzed, non-lipemic) obtained from each individual among those captured multiple times was incorporated into the blood data analyses. Results with a *P*-value <0.05 were considered statistically significant. A Bonferroni adjustment, with an overall significance level of α = 0.05, was used for post hoc comparison of pairs for grouping factors with three or more levels [37].

Statistical tests were also used to assess the effect of covariates on the seroprevalence of pathogen exposure in manatees from Belize. In order to maintain statistical power, these analyses were limited to cases where the sample size of seropositive individuals for a given pathogen exceeded ten manatees in number. Following these guidelines, a Pearson’s chi-square test of independence or Fisher’s exact test was used to evaluate significant differences by sex, age class, season, capture site, and year in relation to serostatus to *L. interrogans* spp.

## Results

Of the 115 manatees for which blood samples were submitted for hematology, biochemistry, or serology, males and females were similarly represented, and age groups were distributed in the same relative proportions within each sex (Table 1). Some individuals (n = 31) were captured multiple times over the 12-year period, resulting in multiple blood samples from these individuals. The majority of manatees were captured in the Southern Lagoon (n = 82), whereas considerably fewer manatees were captured in the Northern Lagoon (n = 8), Placencia Lagoon (n = 6), and Drowned Cayes (n = 19). Based on physical assessment at the time of capture, all individuals included in the study appeared healthy.

**Table 1:** Total number of Antillean manatees (*Trichechus manatus manatus*) included in data results for hematology, biochemistry, and serology in relation to sex and age.^ab^.

Age	Gender	Hematology	Biochemistry	Serology
Adult	Male	28	41	40
	Female	29	34	34
Subadult	Male	8	10	11
	Female	12	15	14
Calf	Male	2	2	2
	Female	3	3	2
Total		82	105	103

a Age classifications are based on standardized total straight length measurements in centimeters: adults >255; subadults 176–225; and calves <176.

b The age classes of 9/112 manatees for which samples were submitted for serologic testing were not available; this included seven males and two females.

In examining sources of variation in mean hematology and biochemistry values, (expressed as estimated mean ± standard deviation), age class comprised the greatest source of variation. Significant parameter differences were also observed in relation to capture location, whereas differences based on sex and season figured less prominently. Accordingly, reference intervals for the majority of blood parameters were reported for the population as a whole (Table 2), with separate reference intervals presented for those blood parameters associated with significant differences by age class (Table 3) and by capture location (Table 4).

**Table 2:** Hematology and biochemistry values for all age classes in wild Antillean manatees (*Trichechus manatus manatus*) in Belize between 1997 and 2009.

Analyte	N	Mean ± SD	Min	Max
MCH (pg)^a^	62	40.0±3.59	28.3	47.3
MCHC (%)^a^	58	31.8±3.28	19.1	42.6
MCV (fl)^a^	36	123.1±22.41	0.0	150.5
Neutrophils (/L)	81	1916.6±990.48	31.0	4472.0
Monocytes (/UL)	81	3.8±17.21	0.0	96.0
Eosinophils (/UL)	81	160.8±277.25	0.0	1333.0
Basophils (/UL)	82	3.9±16.47	0.0	104.0
Glucose (mg/dL)	79	82.1±37.93	43.1	365.0
BUN (mg/dL)^a^	80	5.6±3.91	1.0	17.0
AST (U/L)^a^	69	35.4±16.71	7.0	93.0
ALT (U/L)^a^	69	18.8±14.45	3.0	77.1
GGT (U/L)^a^	79	36.6±11.38	11.0	97.0
Triglycerides (mg/dL)	104	90.2±40.27	0.0	248.0
Albumin (g/dL)	80	4.2±0.89	2.3	6.4
Calcium (mg/dL)	82	10.3±1.19	6.7	14.0
Potassium (mmol/L)	82	5.3±0.70	3.9	7.1
Sodium (mmol/L)	82	151.2±8.81	119.0	171.0
Magnesium (mg/dl)	81	5.2±1.32	2.5	9.3
Chloride (mmol/L)	82	95.8±9.03	73.0	118.0

a MCH  =  mean corpuscular hemoglobin, MCHC  =  mean corpuscular hemoglobin concentration, MCV  =  mean corpuscular volume, BUN  =  blood urea nitrogen, AST  =  aspartate aminotransferase, ALT  =  alanine aminotransferase, GGT  =  γ-glutamyltransferase.

**Table 3:** Hematology and biochemistry values by age class for wild Antillean manatees (*Trichechus manatus manatus*) in Belize between 1997 and 2009 with *P* values^b^ indicating significant age differences.

Analyte	Adults		Subadults		Calves		*P* value^b^
	N	Mean ± SD	N	Mean ± SD	N	Mean ± SD	
Hb (g/dL)^a^	57	10.2±0.99	20	10.8±0.75	5	12.1±2.07	*P* = 0.001
PCV (%)^a^	53	32.8±3.32	19	35.0±2.75	5	39.2±5.90	*P*<0.001
RBC (/L)^a^	42	2.5±0.40	18	2.7±0.22	4	3.2±0.44	*P* = 0.012
WBC (/L)^a^	57	4.6±2.06	20	6.8±2.38	5	13.2±4.40	*P*<0.001
Lymphs (/L)^a^	57	2440.0±1340.07	20	4759.4±2237.55	5	11210.6±4202.28	*P*<0.001
PLT (/L)^a^	47	246.7±109.20	18	344.8±121.22	4	215.5±143.77	*P* = 0.002
Creat (mg/dL)^a^	59	1.6±0.76	19	1.9±0.62	4	2.5±0.52	*P* = 0.032
ALP (U/L)^a^	58	76.7±26.94	19	75.2±23.70	4	130.0±41.34	*P* = 0.011
Chol (mg/dL)^a^	59	111.3±36.83	19	152.2±52.83	4	282.3±48.21	*P*<0.001
TP (g/dL)^a^	58	7.2±0.81	19	6.5±0.70	4	6.8±0.21	*P* = 0.002
Phos (mg/dL)^a^	59	5.2±1.56	19	5.2±1.70	4	9.0±2.49	*P* = 0.001

a Hb  =  hemaglobin, PCV  =  packed cell volume, RBC  =  red blood cell count, WBC  =  white blood cell count, Lymphs  =  lymphocytes, PLT  =  platelet count, Creat  =  creatinine, ALP  =  alkaline phosphatase, Chol  =  cholesterol, TP  =  total protein, Phos  =  phosphorous.

b *P* values from *F*-test with 2 and n–3 *df.*

**Table 4:** Hematology and biochemistry values by capture location for wild Antillean manatees *(Trichechus manatus manatus)* in Belize between 1997 and 2009 with *P* values^bc^ indicating significant capture site differences.

Analyte	Southern Lagoon		Drowned Cayes		Northern Lagoon		Placencia Lagoon		*P* value^bc^
	N	Mean ± SD	N	Mean ± SD	N^d^	Mean ± SD	N^d^	Mean ± SD	
MCV (fl)^a^	19	129.0±7.44	10	121.2±6.19	1	–	2	–	*P* = 0.003^b^
BUN(mg/dL)^a^	58	4.6±2.84	13	10.8±4.56	5	4.1±3.99	4	–	*P*<0.001^c^
ALB (g/dL)^a^	58	4.3±0.92	13	3.9±0.66	5	5.2±0.67	4	–	*P* = 0.035^c^
K (mmol/L)^a^	60	5.1±0.57	13	5.6±0.88	5	6.5±0.44	4	–	*P*<0.001^c^
TG (mg/dL)^a^	75	91.29±39.0	19	85.83±40.53	5	125±53.13	5	55.2±15.87	*P* = 0.048^c^
AST(U/L)^a^	52	33.6±15.81	13	43.9±17.13	1	–	3	–	*P* = 0.021^b^
ALT(U/L)^a^	52	20.3±15.69	13	11.7±6.18	1	–	3	–	*P* = 0.017^b^
GGT(U/L)^a^	57	36.7±8.75	13	28.8±9.79	5	40.7±3.65	4	–	*P* = 0.007^c^

a MCV  =  mean corpuscular volume, BUN  =  blood urea nitrogen, ALB  =  Albumin, K  =  Potassium, TG  =  Triglycerides, AST  =  aspartate aminotransferase, ALT  =  alanine transaminase, GGT  =  gamma-glutamyl transpeptidase.

b *P* values from *t* test with n1+ n2–2 *df*.

c *P* values from *F*-test with 2 and n–3 *df.*

d Sample sizes with <5 observations were not included in the analysis for the blood parameter assessed.

### Hematology

The mean values of several hematology parameters including PCV, RBC, PLT, WBC, and lymphocytes differed significantly in relation to age class (Table 3), while sex had little influence on hematologic indice values. Important interactions between these two covariates were not identified. As with comparisons by sex, mean hematology parameters varied little by capture location and season. The only exceptions to this were mean MCV values, which differed significantly among capture site subpopulations (Table 4), and eosinophil counts, which were signficantly higher during the dry season (264.4±347.32/UL) than during the wet season (49.3±84.04/UL, *P*<0.001).

### Biochemistry

Age class and capture site were both found to be signfiicant sources of variation in several biochemical parameter values (Table 3 and Table 4). In contrast, mean biochemical analyte levels were similar among male and female manatees. With respect to season, only albumin values differed significantly, with higher mean levels found during the dry season (4.7±0.68 g/dL) than during the rainy season (3.8±0.83 g/dL, *P*<0.001).

### Seroprevalence of Selected Pathogens

All manatees sampled in Belize had negative antibody titers to *Brucella* spp., *N. caninum*, *S. neurona*, AIV type A, SLEV, WEEV, and WNV. Seroprevalence results for *Leptospira* spp. established that 23% (26/112) of the sample population was seropositive to at least one serovar of *L. interrogans*. Among those manatees that were seropositive, 14% (16/112) had antibody titers to *L. bratislava*, 4% (5/112) to *L. canicola*, and 4% (5/112) to *L. icterohemorrhagiae.* None of the manatees had positive antibody titers to *L. interrogans* serovars; *grippa*, *hardjo*, and *pomona*. With regard to serologic screening for targeted protozoal organisms, one manatee had an equivocal positive titer to *T. gondii* following the IFAT, but all other individuals were seronegative. On the LAT, 7% (8/112) of manatees had positive antibody titers to *T. gondii*. Serology results for morbillivirus revealed low neutralizing antibody titers to CDV and DMV/PMV in 4% (4/112) of manatees sampled.

In examining serostatus in relation to sex, age class, season, and capture location, we found a significant association between seropositivity to *L. interrogans* and capture site (χ2 = 11.25, *P* = 0.010). Manatees from the Northern Lagoon had the highest prevalence of positive antibody titers to *L. bratislava* (χ2 = 9.99, *P* = 0.010) (Figure 1). Seropositivity to *L. bratislava* was also more prevalent in manatees captured during the dry season (χ2 = 5.36, *P* = 0.021) than during the rainy season. No significant association between serostatus and sex or age class was detected.

## Discussion

Governmental and non-governmental agencies in Belize have made huge strides in manatee conservation over the past two decades; this has been largely achieved through community outreach programs, partnerships with neighboring countries, and dedicated research initiatives. In spite of these steps forward, the overall health status of manatees in this region and the impact of infectious disease on population health have not been well investigated. A few pilot studies have been conducted previously on Antillean manatees to determine hematology and biochemical reference intervals, but sample sizes were limited, and one of these studies was restricted to captive manatees [1], [19], [38], [39]. In the Florida subspecies, normal blood parameters have been documented in healthy free-ranging and captive manatees [27], [40], [41] along with preliminary seroprevalence data [42], [43]. Our study is the first large-scale effort to establish hematology and biochemistry reference intervals in healthy, wild Antillean manatees and report on baseline pathogen exposure in this subspecies.

### Hematology and Biochemistry Parameters

Mean blood values for both hematology and biochemistry parameters differed most significantly in relation to age class. Many of the age differences detected were either similar to the findings in the Florida subspecies [27], [40], or were typical of age-related blood parameter variations observed in other mammals [44], [45]. In the present study, we elected to include calves in comparisons by age in spite of sample size limitations. Establishing health parameters that are age-specific may enhance our present understanding of calf health and assist in determining causes underlying perinatal mortality.

Similar to findings in Florida manatees [40], [44], we found that leukocyte and lymphocyte counts decreased with age, with the highest counts observed in young calves. One finding unique to Belizean manatees, regardless of age, was that neutrophils comprised a much smaller percentage of the total leukocyte count relative to lymphocytes; prior studies have reported the ratio to be relatively equal [40] or often reversed in the case of newborn manatees [40], [44]. In general, leukocyte counts and inflammatory response differ markedly among marine mammals [44]. Manatees typically exhibit a subtle or short-lived rise in leukocytes in response to inflammation, similar to the pattern observed in bovids [46], [47]. Consequently, monitoring relative shifts in manatee leukocyte counts may be more useful than relying on total counts for diagnostic purposes [44]. More recently, serum amyloid A and albumin/globulin ratio have been established as more sensitive markers of inflammation in manatees and are currently being incorporated into health profiles [46], [48].

Significant age differences were also detected in relation to red blood cell indices. Specifically, we established that PCV, Hb, and RBC decreased with increasing age. Overall, this is an unusual trend in terrestrial mammals [45] and was not identified as a significant age-related trend in Florida manatees [40]. Although it does not account for the discrepancy between manatee subspecies, it has been suggested in other marine mammals that higher red blood cell indices in neonates may assist in oxygen regulation as they learn to hold their breath during dives [44]. As shallow water mammals, manatee calves do not have to adapt to deep water dives; however, they may benefit from increased oxygen storage and carrying capacity while learning to remain submerged for increased periods of time. An additional explanation for this finding may be that younger manatees are prone to greater capture stress than adults and consequently experience an epinephrine-type response similar to that reported in horses [45]. Physiologically, a stress reaction of this nature can result in splenic contraction with swift release of erythrocytes into the circulation [45].

With respect to biochemistry parameters, significant age-related trends were detected for TP, cholesterol, ALP, phosphorous, and creatinine. The higher TP levels observed in adult manatees in Belize relative to subadults and calves may be indicative of low grade, chronic inflammation [49], which can be associated with the normal aging process [50], [51]. For all other analytes mentioned above, the highest mean values were observed in calves, followed by subadults and adults, respectively. Similar age-related findings for ALP, cholesterol, and phosphorous were observed in Florida manatees [27]. Physiologically, rapid bone growth in young animals causes elevated levels of ALP and phosphorous [44]. Higher cholesterol levels in calves may be due to nursing and associated milk composition [27], [52] and may also reflect differing rates of lipid catabolism tied to age-dependent developmental requirements [53].

Capture site was a significant source of variation for MCV, BUN, ALB, potassium, triglycerides, AST, ALT, and GGT. However, the biological relevance of these subpopulation differences is limited since all mean parameter values were clinically normal relative to reference intervals established previously in West Indian manatees and in other marine mammals [1], [19], [27], [38], [39], [40], [41], [44]. Most of the differences we identified can probably be explained by subtle variations in diet, water salinity, and other site-specific environmental conditions that differ throughout Belize [54], [55]. It should also be noted that ALT, AST and GGT are not liver-specific in manatees [44], [56] and changes in enzyme levels are therefore not a direct indication of hepato-biliary function. More recent guidelines for diagnosing liver disease in manatees focus on measuring sorbitol dehydrogenase, glutamate dehydrogenase, and tracking bilirubin levels [56], [57].

In accordance with prior research in manatees [27], [38], [39], [40], [41], few significant sex differences were detected among blood parameters, and those that were identified were consistent with normal, healthy levels reported in other manatee populations. Variation in blood parameters by season was also uncommon, but we found that albumin levels were significantly lower during the rainy season than during the dry season. This variation might be related to changes in hydration status stemming from seasonal alterations in water levels and associated shifts in salt and fresh water availability [17]. Additionally, we observed higher eosinophil counts during the dry season compared to the wet season, which may reflect shifts in parasite load following heavier periods of rainfall [58], [59].

### Pathogen Exposure

Routine screening for common marine mammal pathogens in manatees from Belize, and more broadly, monitoring for emerging disease trends across sirenian populations, are essential given the vulnerability of manatees and dugongs worldwide. In comparing our findings to seroprevalence results reported in the 1996 Florida manatee study [42], several significant differences should be highlighted. First, 7% of Florida manatees were seropositive to *Brucella* spp. [42], whereas none of the manatees sampled in Belize had positive antibody titers to this pathogen. Additionally, a single manatee in the Florida study tested positive for exposure to avian influenza [42]. Another distinction is that exposure to protozoal agents, morbillivirus, and WNV [60] were not assessed in the 1996 survey of the Florida subspecies [42].

Aside from the aforementioned differences, seroprevalence findings were relatively similar in both populations. Like manatees from Belize, all of the Florida manatees had negative titers to SLEV and WEEV [42]. Additionally, seroprevalence to *Leptospira* spp. was found in comparable levels in both subspecies. Overall, 22% of Florida manatees [42] and 23% of manatees from Belize had positive antibody titers to one or more serovars of *L. interrogans*. A third study investigating leptospirosis exposure in captive Amazonian manatees (*Trichechus inunguis*) in Brazil recently established that 31% of the sample population (n = 74) was seropositive to *L. interrogans spp.* [61] and serves as a further comparison.

Among those individuals with positive leptospirosis titers in Belize and Florida, many were seropositive to multiple serovars. In contrast, this was reported to be the case with only one leptospirosis-positive manatee in Brazil [61]. In the present study, to avoid ambiguities resulting from possible cross-reaction among *L. interrogans* serovars, only the serovar with the highest titer was considered positive. This approach, which assumed that an individual was unlikely to be exposed to more than one serovar may be overly conservative, as multiple seroprevalences to *L. interrogans* serovars have been reported in other marine mammals [62]. Evaluating studies side-by-side like this has the potential to offer insight on emerging trends; however, differences in testing methods, serovar panels, and interpretation of assay results preclude the ability to make direct comparisons about pathogen exposure status between sirenian populations. Consequently, those engaged in manatee disease research should seek to establish uniform assay and reference laboratories that can apply standard methodology in diagnostic techniques and interpretation of test results.

In spite of relatively low seroprevalence of pathogen exposure in both Belize and Florida manatee populations, disease remains a threat. Indications of lowered immunity and increased susceptibility to infection in the Florida subspecies became apparent with the discovery of a novel, manatee-specific papillomavirus in 1997 [43], [63], [64]. In general, the number of infectious agents discovered in marine mammals has risen over the past two decades [10], [65]. Anthropogenic drivers are likely responsible for many of the factors contributing this trend. Weakened immunity and/or increased exposure risk may arise from climatic alterations, depletion of food sources through over-fishing, and exposure to chemical byproducts and pathogen waste from terrestrial runoff [10], [12], [13], [66]. In turn, the risk of interspecies disease transmission increases as population growth and landscape change push humans, domestic animals, and wildlife in closer proximity [11], [12], [13]. In combination, these stressors place marine species living close to the land-water interface at heightened risk of infection.

As nearshore species, with regular movement between fresh and salt water environments [67], [68], manatees may be susceptible to infection through a variety of means including contaminated land-water runoff, contact with other nearshore species [69], and arthropod-borne infections contracted when feeding on shoreline vegetation [22]. As important pathways of disease transmission are determined and high risk zones identified, preventive measures may be developed with added focus given to those pathogens that were associated with low levels of seropositivity in the manatee population of Belize. A preliminary discussion of these pathogens is given in the section that follows.

### Leptospirosis

Maintained in a variety of wild and domestic animal hosts, leptospirosis poses a serious human health risk worldwide [70], [71]. While there have been no case reports of leptospirosis in manatees around Belize, almost a quarter of the manatees captured in this study were seropositive to *L. bratislava*, *L. canicola*, and/or *L. icterohemorrhagiae.* Clinical infection with *Leptospira* spp. has been confirmed in several species of pinniped [72], [73], but is particularly well documented in California sea lions [74]. Cyclical epizootics in this species tend to follow El Niño years [74], suggesting that environmental drivers may influence pathogen or host dynamics [71], [73], [74]. Similar epidemiologic and ecological factors may give rise to outbreaks of *L. interrogans* spp. in manatees around Belize. The potential for high die-offs should an epizootic occur warrants routine serologic screening of this pathogen in manatees of this region as well as examination for characteristic lesions and relevant histopathology performed on all carcasses recovered for necropsy.

**Figure 2:**
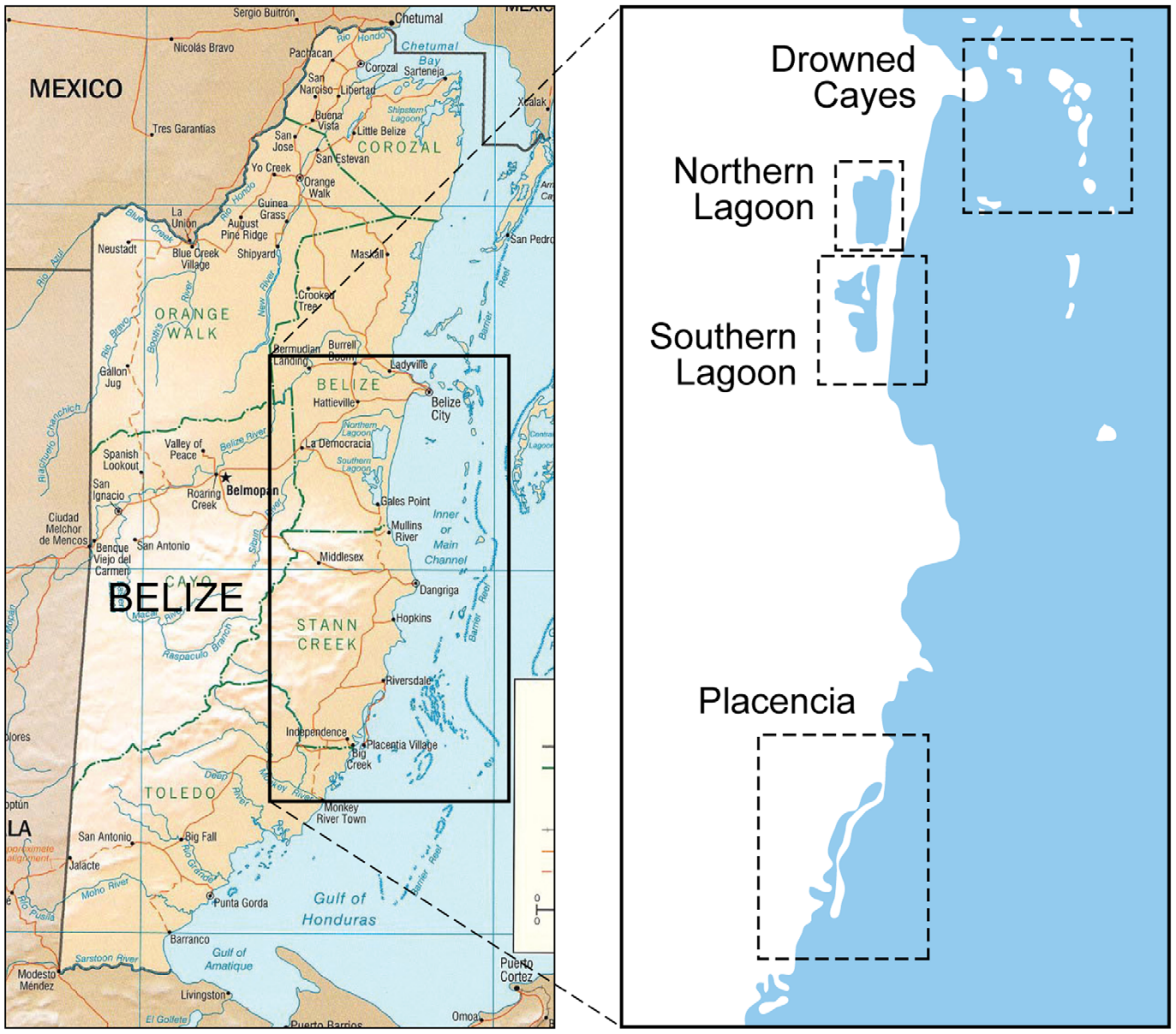
Map of four primary study sites in Belize where capture and release of free-ranging manatees occurred between 1997 and 2009.

A significantly greater proportion of manatees captured in the Northern Lagoon were seropositive to *L. interrogans* spp. compared to the other capture sites; this association was most pronounced for *L. bratislava* exposure. Prevalence levels were similar among manatees from the Southern and Placencia lagoons, whereas all manatees sampled from the Drowned Cayes were seronegative. Potential reasons for the higher exposure levels in the Northern Lagoon may be due to the fact that it is more land-locked than the other capture locations (Figure 2). Consequently, it may be subject to greater water stagnancy, preventing quick removal of the pathogen from the environment through regular water turnover [75]. Given its geography, there may also be greater potential for exposure to infection from terrestrial reservoir hosts via land-water runoff. A seasonal association with seroprevalence to leptospirosis was also identified, with a significantly higher percentage of *L. bratislava*-positive manatees detected during the dry season than during the wet season. However, we could not investigate the effect of season on the incidence of infection since serology only provides evidence of exposure at some unknown time in the past. Moreover, wet and dry seasons were too brief in duration to infer incidence based on changing titer levels of individuals that were captured multiple times.

### Toxoplasma Gondii

*Toxoplasma gondii* has been documented as a cause of high morbidity and mortality in a variety of marine mammals [29], [76], [77], [78], [79], [80], [81], [82] and poses a serious human health risk to immunocompromised individuals and children infected in utero [83]. Felidae serve as the definitive hosts for *T. gondii* [84], and in southern sea otters, both feral and domestic cats have been implemented in the transmission pathway by shedding oocysts in reservoirs and other water sources [76]. Similarly, transport of oocysts from land-based effluent may be a source of exposure to *T. gondii* in manatees [76], especially given their occasional reliance on sewage effluents as fresh water drinking sources [85]. Despite natural behaviors such as this that may increase their exposure risk to *T. gondii*, manatees in Belize had a low prevalence of seropositivity to this zoonotic protozoan. Among the eight manatees in Belize that were seropositive for *T. gondii* on the LAT, none had titers that exceeded 1∶320. Four of these seropositive manatees were sampled multiple times, but the magnitude of difference between serial titers was either too small, or the time elapsed between serial titers too long, to make valid inferences about the timing of infection or seroreversion.

Serology data on *T. gondii* in other manatee populations is limited, but a recent study in captive Amazonian manatees in Brazil reported a 39.2% seroprevalence to *T. gondii* [61]. A partial explanation for the lower exposure levels in manatees from Belize may be related to the fact that domestic cats are infrequently observed on shoreline beaches in the region. However, jaguar tracks are commonly sited at the water’s edge, underscoring the importance of regular screening and increased focus on identifying probable sources of contamination. Additional studies of *T. gondii* in other manatee populations are currently underway, but to date there have only been two isolated cases of the protozoan documented in West Indian manatees; one of these cases was described in an Antillean manatee from Guyana [77], a second case was reported in a Florida manatee that died from complications of menigoencephalitis [85]. A concerted effort to collect histologic samples from beached carcasses and obtain serial titers on suspect cases of *T. gondii* in captive manatees will assist in efforts to establish a gold standard and common testing methodology and will aid in establishing the overall impact of this protozoan on manatee population health.

### Morbillivirus

In the late 1980s and early 1990s, four novel morbilliviruses were traced to mass mortalities in several pinniped and cetacean populations across northwestern Europe, Siberia, and the Mediterranean [33], [86]. Among the morbilliviruses implicated in the outbreak, phocine distemper virus (PDV), and the more closely related porpoise morbillivirus (PMV) and dolphin morbillivirus (DMV) are believed to be endemic in certain marine mammal communities [33]. However, it is thought that heavy losses can occur when these pathogens are introduced into naïve populations [86]. As demonstrated in Mediterranean monk seals (*Monachus monachus*) [87], such a scenario has the potential to devastate endangered species, like manatees, which have low reproductive rates, heavy maternal investment, and long-life spans [69].

Although manatees around Belize coexist with several cetacean species, we found minimal serologic evidence of exposure to morbillivirus in the manatees we sampled. Of the 112 manatees tested, only one adult female manatee and three adult males had low positive antibody titers against CDV and PMV/DMV. Among the four manatees that were seropositive to morbillivirus, antibody titers were slightly higher against PMV/DMV than against CDV, but the low neutralization responses overall (i.e., no titer >1∶20) were not sufficient enough to suggest that a morbillivirus of cetacean origin was the likely source of infection. Similar seroprevalence results for morbillivirus were published on Florida manatees in 1995 in which 4% of the sample population (n = 148) was reported to be seropositive [69]. Neutralization titers against DMV and PMV were slightly higher in Florida manatees than those reported in the present study and led researchers to hypothesize that bottlenose dolphins may have transmitted the infection [69]. In the same survey, a small number of wild manatees from Guyana and hand-reared Amazonian manatees were also screened, and all individuals tested were found to be seronegative [69].

Morbillivirus has yet to be isolated from a manatee, and reports of suspect cases have not been documented in the species [69]. Investigators from the Florida study speculated that the presence of low positive titers may indicate that either viral replication is inadequate to cause clinical signs of infection or that manatees cohabitate in numbers too small to enable adequate host-host transmission to elicit an outbreak [69]. However, given the devastating losses suffered in previously unexposed pinniped and cetacean populations, and the fragility of manatee populations in general, regular testing for morbillivirus in health assessments and continued monitoring for clinical cases is crucial.

Understanding how disease may affect population growth in Antillean manatees from Belize is especially important at present since coastal development is increasingly taxing the resilience of this nearshore mammalian species. Human encroachment has collectively destroyed important seagrass beds and resulted in the clearance of vital mangrove areas [2]. Subsequent changes in water quality and food abundance not only place constraints on the manatees of this region, but also challenge the robustness of other organisms that inhabit these waters [2], [9]. Antillean manatees may be well suited as sentinel species in this regard [7], [9], [10]. As such, the importance of cause-specific mortality studies and disease surveillance in healthy populations of this species must be emphasized.

Although low prevalence of pathogen exposure was detected in the manatee population from Belize, supplemental mortality data and improved testing methodologies are needed to gain a better understanding of the current situation. These next steps are crucial as a single disease outbreak in an immunologically naïve, seronegative population could cause epidemic mortality and substantially impede recovery [12], [88]. Given this possibility, supporting long-term research that expands on the findings presented here, may be critical to the success of Belize’s manatee recovery efforts and assist in safeguarding the health and conservation of the surrounding ecosystem.
